# A Comparison of Hepatocyte Cytotoxic Mechanisms for Docetaxel and PLGA-Docetaxel Nanoparticls

**Published:** 2017

**Authors:** Bahram Daraei, Marjan Aghvami, Jalal Pourahmad, Rassoul Dinarvand

**Affiliations:** a *Department of Toxicology, Faculty of Medical Sciences, Tarbiat Modares University, Tehran, I.R. of Iran. *; b *Department of Toxicology, Faculty of Medical Sciences, Tarbiat Modares University, Tehran, I.R. of Iran. *; c *Department of Toxicology & Pharmacology, Faculty of Pharmacy, Shaheed Beheshti University of Medical Sciences, Tehran, I.R. of Iran. *; d *Medical Nanotechnology Research Center Address: Faculty of Pharmacy, Tehran University of Medical Sciences, Tehran, I.R. of Iran.*

**Keywords:** Docetaxel, Polylactide-co-glycolide, PLGA-DTX nanoparticles, Cytotoxicity, Rat Hepatocyte, Silymarin

## Abstract

Docetaxel (DTX) is one of the most widely used drugs in oncology due to its high efficacy against several cancers. Though, its routine clinical administration, formulated in tween 80, causes serious side effects. Polylactide-co-glycolide (PLGA), biodegradable polyester synthesized and approved for human use, is employed to overcome these problems.

In this investigation, we compare the cytotoxic mechanisms of DTX and PLGA-DTX in isolated rat hepatocytes. Cytotoxicity of DTX and PLGA-DTX were associated with reactive oxygen species formation, activation of caspases cascade, collapse of mitochondrial membrane potential (MMP), lysosomal membrane leakiness and ATP depletion. Our results also showed that CYP2E1 is involved in the oxidative stress cytotoxicity mechanism and both drugs are detoxiﬁed via phase II metabolic methylation. Furthermore, we concluded that PLGA-DTX is bioactivated by GSH. It could also potentiate hepatocyte toxicity through a mitochondrial/lysosomal toxic cross-talk. In addition to these observed differences, it is likely that mode of hepatocyte membrane penetration is different between these compounds.

## Introduction

Docetaxel (N-debenzoyl-N-tert butoxycarbonyl-10-deacetyl) is a semi-synthetic derivative of the taxoid family of anticancer agents. Same as paclitaxel, this substance is extracted from the European yew tree (*Taxus baccata L*.) (1). This class of anticancer agents stabilizes microtubules by binding to β-tubulin, thereby cause cell-cycle arrest and apoptosis (2). Docetaxel is a highly effective medicine approved for treatment of common cancers, but its effectiveness in clinical practice is downgraded by severe side effects (3). All anti-neoplastic drugs damage mechanisms that are essential to cell growth and docetaxel, like many others, prevents new cells formation, as well as, cell maturation and multiplication. However, none of these end points is specifically targeted to be achieved in the tumor cells merely, but healthy cells may also be adversely affected, resulting in drug-related side effects (4). On the other hand, since docetaxel is insoluble in water, tween 80 is usually used as a co-solvent in its pharmaceutical formulations as well. Unfortunately, tween 80 may cause anaphylaxis in sensitive patients. Thus, a large number of anti-allergic agents, like glucocorticoides, are usually included in chemotherapeutic protocols. Moreover, chemotherapy with docetaxel has a variety of side effects, just as peripheral neurotoxicity, bone marrow depression, and fluid retention (5). 

To reduce all these side effects, an alternative nanoparticle-based drug delivery system can be a solution. For this purpose, a number of polymers have recently been investigated as biodegradable loading vehicle for newly designed docetaxel nanoparticle formulations in cancer chemotherapy. The most widely used class of biodegradable and biocompatible polymers, approved by Food and Drug Administration (FDA), is poly (lactic acid) (PLA), poly (glycolic acid) (PLGA), and their copolymers (6). PLGA-encapsulated docetaxel provides a satisfactory sustained drug release (7) and reduces the non-specific targeting of non-tumoral cells, resulting in significant decrease of undesirable side effects (8). Moreover, it is also reported that nanoparticles anticancer formulations showed higher cytotoxic effects than free drugs, via different mechanisms. First of all, free docetaxel molecules, which are transported into the cytoplasm by passive diffusion, are effluxed out of the cell by P-glycoprotein (P-gp) pumps while NPs are taken up through an endocytosis pathway, enabling them to escape from the effect of P-gp pumps, resulting in a higher cellular concentration of the entrapped drug (9). Besides, NPs were adsorbed on the cell surface leading to an increase in the drug concentration near the cell membrane (10). 

Moreover, *in-vitro* studies indicated that docetaxel is primarily metabolized and thus eliminated by cytochrome 3A4 in liver. Co-administration of drugs and substances which modulate the activity of CYP3A4 is, therefore, likely to have desirable clinical consequences (11). Silymarin is one of these substances, which is commonly used for its hepatoprotective and antioxidant properties and recently it is under study in cancer research both as a chemopreventive and as an adverse effects modulating compound (12). It was shown that silymarin decreases the expression and activity of CYP3A family in human hepatocyte cultures (13), in this way, when it used in combination with free docetaxel or its nano form it may have a protective effector not respectively. Previous studies have shown that liver is the main organ for docetaxel metabolism via hepatic CYP3A isozymes (12, 14). Besides, liver is one of the target sites for docetaxel toxicity. The intracellular pathways of docetaxel or its nanoparticle formulations induced hepatotoxicity are not well investigated. Additionally, there are only few studies in the literature about the mechanisms of cellular and molecular toxicity of docetaxel (6, 15, 16). Many observations have demonstrated that nanomaterials could lead to the increased formation of reactive oxygen species (17) either through mitochondrial dysfunction, or cellular redox cycling. ROS can also be produced by the NADPH oxidase or as a product of cytochrome P450(s) metabolism (18). 

Based on these investigations we assumed that the cytotoxicity of PLGA-loaded docetaxel may involve metabolic activation and propagation of reactive oxygen species. Hence, in the present study, we have investigated and compared the cytotoxic mechanisms of PLGA-loaded docetaxel and docetaxel alone in freshly isolated rat hepatocytes. 

## Materials and methods

*Chemicals*

Docetaxel (Cipla ,India),1-Bromoheptane, silymarin and rhodamine 123 were obtained from Aldrich Chemical Company (Milwaukee, WI, USA). Collagenase (from *Clostridium histolyticum*), 2′,7′ dichlorofluorescin diacetate (DCFH-DA), bovine serum albumin (BSA) and HEPES (4-(2-hydroxyethyl)-1-piperazine ethanesulfonic acid were purchased from Boehringer–Mannheim (Montreal, Canada). O-phthalaldehyde (OPT), N-ethylmaleimide (NEM), butylated hydroxytoluene (BHT), cyclosporine, diphenyliodoniumchloride, reduced and oxidized glutathione (GSH and GSSG) Trypan blue, D-mannitol, dimethyl sulfoxide (DMSO), trichloroacetic acid (TCA), Ketamine, Xylazine, heparin and the other chemicals were obtained from Sigma (St. Louis, MO, USA). Acridine orange and dichlorofluorescin diacetate were purchased from Molecular Probes (Eugene, Ore, USA). All chemicals were of the highest commercial grade available. PLGA-DTX was prepared in the Faculty of Pharmacy, Tehran University of Medical Sciences. Physicochemical properties of the drug-loaded nanoparticles (NPs) were previously investigated by its manufacturers (15). They measured particle size, size distribution, polydispersity, and zeta potential of the NPs by laser light scattering. Additionally scanning electron microscopy (SEM) was used to determine the shape and surface morphology of the NPs. Under SEM observation all NPs were found to have fine spherical shape with a relatively monodispersed size distribution. The main characteristics of the PLGA-DTX NPs are listed as follow: 

Particle size: 175 ± 13 nm

Polydispersity index < 0.15

Zeta potential < – 12.2 ± 0.6 mV

*Animals*

Male Sprague–Dawley rats weighing 280 to 300 g were used in the study, provided by Razi Vaccine and Serum Research Institute (Karaj, I.R. Iran). All rats were housed in a room at a constant temperature of 25 °C on a 12/12 h light/dark cycle with food and water available ad libitum. All experiments were conducted according to the ethical standards and protocols approved by the Committee of Animal Experimentation of Tarbiat Modares University, Tehran, I.R. Iran. 

*Isolation and incubation of hepatocytes*

Hepatocytes were obtained by collagenase perfusion of the liver as described by Moldeus (19). Cells were suspended at a density of 10^6^ cells/mL in round-bottomed flasks rotating in a water bath maintained at 37^ o^C in Krebs–Henseleit buffer (pH = 7.4), supplemented with 12.5 mM HEPES under an atmosphere of 10% O_2_, 85% N_2_, and 5% CO_2_. Each flask contained 10 mL of hepatocyte suspension. Hepatocytes were preincubated for 30 min prior to addition of chemicals. Stock solutions of all chemicals were prepared fresh prior to use. To avoid either non toxic or very toxic conditions in this study, we used EC_50_2-h concentration for DTX (6 nM) and PLGA-DTX (3 nM) in the isolated hepatocytes. In order to determine this value for the investigated compound, dose–response curves were plotted and then EC_50_ was determined based on a regression plot of three different concentrations (19). In general the hepatocytes were incubated with DTX or PLGA-DTX for 3 h and all the pretreatments were added 15 min before drug addition. GSH-depleted hepatocytes were prepared by using 1-bromohepatane as described by Khan and O’Brien (20).

*Cell viability*

The viability of isolated hepatocytes was assessed from the integrity of the plasma membrane as determined by the trypan blue (0.2% w/v) exclusion test (19). Aliquots of the hepatocyte incubate were taken at different time points during the 3 h incubation period. At least 80–90% of the control cells were still viable after 3 h.

*Determination of reactive oxygen species*

To determine the rate of hepatocyte reactive oxygen species generation induced by DTX & PLGA-DTX, dichlorofluorescin diacetate (DCFHDA) (1.6 μM) was added to the hepatocytes. It penetrates hepatocyte cells and becomes hydrolyzed to non-fluorescent dichlorofluorescin. The latter then reacts with reactive oxygen species to form the highly fluorescent dichlorofluorescein (DCF), which effluxes the cell. The fluorescence intensity of DCF was measured using a Shimadzu RF5000U fluorescence spectrophotometer. Excitation and emission wavelengths were 500 and 520 nm, respectively. The results were expressed as fluorescent intensity per 10^6^ cells(21).

*Intracellular GSH and extra cellular GSSG assessment*

GSH and GSSG were determined according to the spectrofluorometric method (22). Each sample was measured in quartz cuvettes using a fluorimeter set for 350 nm excitation and 420 nm emission wavelengths.

*Mitochondrial membrane potential assay*

Mitochondrial uptake of the cationic fluorescent dye, rhodamine 123 (1.5 μM), has been used for estimation of mitochondrial membrane potential. The amount of rhodamine 123 remaining in the incubation medium was measured fluorimetrically using a Shimadzu RF5000U fluorescence spectrophotometer set at 490 nm excitation and 520 nm emission wavelengths. The capacity of mitochondria to take up the rhodamine 123 was calculated as the difference between control and treated cells in rhodamine 123 fluorescence. Our data were demonstrated as the percentage of mitochondrial membrane potential collapse (%ΔΨm) in all treated (test) hepatocyte groups (23).

*Lysosomal membrane integrity assay*

Hepatocyte lysosomal membrane stability was determined from the redistribution of the fluorescent dye, acridine orange. Aliquots of the cell suspension (0.5 mL) that were previously stained with acridine orange (5 µM) were separated from the incubation medium by 1 min centrifugation at 1000 rpm. The cell pellet was then resuspended in 2 mL of fresh incubation medium. This washing process was carried out for two times to remove the fluorescent dye from the media. Acridine orange redistribution in the cell suspension was then measured fluorometrically using a Shimadzu RF5000U fluorescence spectrophotometer set at 495 nm excitation and 530 nm emission wavelengths. Lysosomal membrane damage was determined as difference in redistribution of acridine orange from lysosomes into cytosol between treated cells and control cells. Our data were demonstrated as the percentage of lysosomal membrane leakiness in all treated (test) hepatocyte groups (24).

*Determination of Caspase-3 Activity*

Caspase-3 activity was determined in cell lysate of hepatocytes from different treatments using Sigma’s caspase-3 assay kit (CASP-3-C) (25). In brief, this colorimetric assay is based on the hydrolysis of substrate peptide, Ac-DEVD-ρNA, by caspase-3. The released moiety (ρ-nitroaniline) has a high absorbance at 405 nm. The concentration of the ρ-nitroaniline (µM) released from the substrate is calculated from the absorbance values at 405 nm or from a calibration curve prepared with defined ρ-nitroaniline solutions. 

*Determination of apoptosis*

Apoptotic cell death is accompanied by a change in plasma membrane structure by surface exposure of phosphatidylserine (PS), while the membrane integrity remains unchallenged. Surface exposed PS can be detected by its affinity for annexin V, a phospholipid binding protein. For the quantification of annexin V-positive apoptotic cells, flow cytometry can best be applied using a single cell suspension prepared from the cells or tissue under examination (26). Apoptosis was determined using *BioVision* Annexin V-FITC Apoptosis Detection Kit. The kit also can differentiate between apoptosis and necrosis when performing both Annexin V-FITC and PI staining. Viable cells will contain neither stain. Cells in apoptosis with intact plasma membrane integrity are stained only by annexin V-FITC, whereas cells in secondary necrosis, the phase consecutive to apoptosis in vitro, contain both stains.

*Determination of cellular energy status*

ATP was measured luminometrically based on luciferin–luciferase bioluminescence reaction (27). Briefly, the reaction is catalyzed by the enzyme luciferase obtained from the firefly (*Photinus pyralis*). The Mg^2+^ /ATP converts the luciferin into a form which is capable of being catalytically oxidized by the luciferase in a high quantum yield chemiluminescent reaction, according to the following equation (28):

ATP + D-luciferin + O_2 _luciferase oxyluciferin + AMP + PPi + light

Cellular ATP was measured by direct lysis of the cells with suitable detergent; in this case the released ATP reacts with the luciferin-luciferase and produces light with a peak emission at 560 nm. 

The intensity of light is proportional to the amount of ATP in the reaction mixture and was measured by Berthold FB12 Luminometer.

*Statistical analysis*

Levene›s test was used to check the homogeneity of variances. Data were analyzed using one-way analysis of variance (ANOVA) followed by Tukey›s HSD as the post hoc test. Results were presented as mean ± S.D. of triplicate samples. The minimal level of significance chosen was P<0.05.

## Results

As shown in Table 1. PLGA-DTX was more effective than DTX in causing hepatocyte membrane lysis as determined by trypan blue uptake. In addition, when hepatocytes were incubated with DTX or PLGA-DTX at these EC_50_ concentrations, ROS formation determined by the oxidation of DCFH to DCF was significantly (P < 0.05) increased. However, PLGA-DTX was more powerful than DTX at inducing ROS formation (Table 1).

Interestingly, PLGA-DTX-induced cytotoxicity and ROS generation was potentiated by GSH and trifluoperazine (Table 1); however, in this case DTX did not show any significant effect on hepatocyte cytotoxicity and ROS formation. Depleting hepatocyte methyl groups by hypomethylator such as DMSO significantly increased both DTX and PLGA-DTX cytotoxicity and ROS formation. All the pretreatments did not show any significant changes on the cytotoxicity and ROS generation at concentrations used when incubated alone in hepatocytes (data not shown). Depleting hepatocyte GSH beforehand potentiated DTX-induced cytotoxicity although this process caused cellular protection against PLGA-DTX toxicity.

**Table 1:** Effect of ROS scavengers, MPT pore-sealing agents & lysosomotropic agents on DTX and PLGA-DTX induced hepatocyte lysis & ROS formation

Treatment Group	Addition	Cytotoxicity (3 h)	DCF (1 h)
Control	**-**	21 ± 2	106 ± 5
Nano formulation of DTX	PLGA-DTX(3 nM)	75 ± 4*	293 ± 15*
+Antioxidants^a^	+Mannitol (50 mM)	49 ± 5†	140 ± 7†
	+BHT (50 M)	45 ± 5†	138 ± 7†
+MPT pore-sealing Agents^a^	+Carnitine (2 mM)	51 ± 5†	152 ± 8†
	+Cyclosporin (2 M)	50 ± 3†	149 ± 7†
+Lysosomotropic Agents^a^	+Choloroquine (100 M)	39 ± 4†	128 ± 6†
	+Methylamine (30 mM)	43 ± 3†	127 ± 6†
	+ Monensin (10 M)	45 ± 3†	123 ± 6†
+ NADPH-P450 reductase inhibitor^a^	+Diphenyliodonium chloride (50 M)	28 ± 3†	107 ± 5†
+ CYP2E1 inhibitors	+Phenylimidazole (300 M)	46 ± 5†	129 ± 6†
	+ 4-Methylpyrazole (500 M)	44 ± 4†	126 ± 6†
+ CYP3A4 inhibitors	+ Troleandomycin (10 M)	84 ± 3†	297 ± 10†
	+ Ketoconazole (10 M)	80 ± 3†	288 ± 10†
+ Hepatoprotectant	+Silymarin (100 M)	34 ± 4†	120 ± 6†
+ Methyl Donors a	+Methionine (1 mM)	34 ± 3†	127 ± 6†
	+Folic acid (100 M)	32 ± 3†	123 ± 6†
	+Betaine (2 mM)	39 ± 5†	130 ± 7†
+ Hypomethylator a	+DMSO (150 M)	92 ± 5†	360 ± 10†
	+ GSH (5 mM)	93 ± 4†	394 ± 15†
+ GSH synthesis stimulator a	+Trifluoperazine (15 M)	86 ± 3†	312 ± 16†
+GSH depleting agent a	+1-Bromoheptane	39 ± 4†	151 ± 7†
Free Drug	DTX (6 nM)	72 ± 4*	277 ± 14*
+Antioxidants^a^	+Mannitol (50 mM)	30 ± 3‡	140 ± 7‡
	+BHT (50 M)	48 ± 5‡	134 ± 7‡
+MPT pore-sealing Agents^a^	+Carnitine (2 mM)	48 ± 5‡	156 ± 8‡
	+Cyclosporin (2 M)	45 ± 5‡	152 ± 8‡
+Lysosomotropic Agents^a^	+Choloroquine (100 M)	48 ± 5‡	142 ± 7‡
	+Methylamine (30 mM)	50 ± 3‡	105 ± 5‡
	+ Monensin (10 M)	53 ± 4‡	302 ± 15‡
+ NADPH-P450 reductase inhibitor^a^	+Diphenyliodonium chloride(50 M)	26 ± 3‡	93 ± 5‡
+ CYP2E1 inhibitors^a^	+Phenylimidazole (300 M)	52 ± 3‡	260 ± 13‡
	+ 4-Methylpyrazole (500 M)	50 ± 4‡	256 ± 15‡
+ CYP3A4 inhibitors^a^	+ Troleandomycin (10 M)	93 ± 3‡	302 ± 5‡
	+ Ketoconazole (10 M)	98 ± 4‡	308 ± 5‡
+ Hepatoprotectant	+Silymarin (100 M)	63 ± 3‡	269 ± 13‡
+ Methyl Donors a	+Methionine (1 mM)	45 ± 5‡	136±7‡
	+Folic acid (100 M)	40 ± 4‡	130±7‡
	+ Betaine (2 mM)	42 ± 4‡	134±7‡
+ Hypomethylator a	+DMSO (150 M)	89 ± 4‡	281±14‡
	+ GSH (5 mM)	48 ± 5‡	115±6‡
+ GSH synthesis stimulator a	+Trifluoperazine (15 M)	40 ± 3‡	126±6‡
+GSH depleting agent a	+1-Bromoheptane	80 ± 3‡	205±10‡

Hepatocytes (10^6^ cells/mL) were incubated in Krebs–Henseleit buffer pH =7.4 at 37^ oC^ for 3.0 h following the addition of PLGA-DTX. Cytotoxicity was determined as the percentage of cells that take up trypan blue [Moldeus et al.,1978] dichlorofluorescein (DCF) formation was expressed as fluorescent intensity units [Shen et al., 1996]. GSH-depleted hepatocytes were prepared as described by Khan and O’Brien [Khan and O'Brien 1991]. Values are expressed as mean ± SD of three separate experiments (n = 3).

PLGA NPs were not cytotoxic at all concentrations tested (data not shown).

a All these agents did not show any toxic effect on hepatocyte at concentrations used (data not shown).

* Significant difference in comparison with control hepatocytes (P<0.05).

† Significant difference in comparison with PLGA-DTX treated hepatocytes (P<0.05).

‡ Significant difference in comparison with DTX treated hepatocytes (P<0.05).

**Table 2:** Mitochondrial membrane potential decline during PLGA-DTX and DTX induced hepatocyte injury

**Treatment Group**	**Addition**	**%ΔΨm**		
		**Incubation time**		
		**15 min**	**30 min**	**60 min**
Nano formulation of DTX	PLGA-DTX (3nM)	63 ± 3	67 ± 3	70 ± 4
+Antioxidants^a^	+Mannitol (50 mM)	24 ± 2*	27 ± 3*	29 ± 3*
	+BHT (50 M)	26 ± 3*	28 ± 3*	30 ± 3*
+MPT pore-sealing Agents^a^	+Carnitine (2 mM)	25 ± 3*	28 ± 3*	35 ± 4*
	+Cyclosporin (2 M)	22 ± 2*	26 ± 3*	33 ± 3*
+Lysosomotropic Agents^a^	+ Chloroquine (100 M)	29 ± 3*	31 ± 3*	35 ± 4*
	+ Methylamine (30 mM)	34 ± 3*	36 ± 4*	40 ± 4*
	+ Monensin (10 M)	32 ± 3*	34 ± 4*	43 ± 4*
+ NADPH-P450 reductase inhibitor^a^	+Diphenyliodonium chloride (50 M)	26 ± 3*	30 ± 3*	38 ± 4*
+ CYP2E1 inhibitors^a^	+Phenyl Imidazol (300 M)	27 ± 3*	28 ± 3*	30 ± 3*
	+ 4-Methylpyrazole (500 M)	25 ± 4*	30 ± 5*	34 ± 5*
+ CYP3A4 inhibitors^a^	+ Troleandomycin (10 M)	75 ± 4*	79 ± 4*	87 ± 4*
	+ Ketoconazole (10 M)	74 ± 4*	80 ± 4*	86 ± 4*
+ Hepatoprotectant	+Silymarin (100 M)	27 ± 3*	30 ± 3*	32 ± 3*
+ Methyl Donors a	+Methionine (1 mM)	28 ± 3*	29 ± 3*	31 ± 3*
	+Folic acid (100 M)	30 ± 3*	32 ± 3*	33 ± 3*
	+Betaine (2 mM)	33 ± 3*	35 ± 4*	36±4*
+ Hypomethylator a	+DMSO (150 M)	79 ± 4*	83 ± 4*	88 ± 4*
	+ GSH (5 mM)	76 ± 4*	79 ± 4*	85 ± 4*
+ GSH synthesis stimulator a	+Trifluoperazine (15 M)	70 ± 4*	77 ± 4*	83 ± 4*
+GSH depleting agent a	+1-Bromoheptane	31 ± 3*	38 ± 4*	40 ± 4*
Free Drug	DTX(6 nM)	56 ± 3*	59 ± 3*	62 ± 3*
+Antioxidants^a^	+Mannitol (50 mM)	26 ± 3†	28 ± 3†	31 ± 2†
	+BHT (50 M)	20 ± 2†	22 ± 2†	23 ± 2†
+MPT pore-sealing Agents^a^	+Carnitine (2 mM)	40 ± 3†	46 ± 3†	56 ± 3†
	+Cyclosporin (2 M)	42 ± 3†	48 ± 3†	58 ± 4†
+Lysosomotropic Agents^a^	+ Chloroquine (100 M)	56 ± 3†	58 ± 3†	63 ± 3†
	+ Methylamine (30 mM)	54 ± 3†	57 ± 3†	61 ± 3†
	+ Monensin (10 M)	59 ± 4†	63 ± 4†	76 ± 4†
+ NADPH-P450 reductase inhibitor^a^	+Diphenyliodonium chloride (50 M)	42 ± 4†	48 ± 5†	53 ± 3†
+ CYP2E1 inhibitors^a^	+Phenyl Imidazol (300 M)	11 ± 1†	14 ± 1†	17 ± 2†
	+ 4-Methylpyrazole (500 M)	13 ± 3†	17 ± 3†	21 ± 4†
+ CYP3A4 inhibitors^a^	+ Troleandomycin (10 M)	65 ± 5†	71 ± 3†	87 ± 3†
	+ Ketoconazole (10 M)	64 ± 4†	70 ± 5†	81 ± 3†
+ Hepatoprotectant	+Silymarin (100 M)	48 ± 4†	52 ± 4†	60 ± 5†
+ Methyl Donors a	+Methionine (1 mM)	29 ± 3†	31 ± 3†	32 ± 3†
	+Folic acid (100 M)	26 ± 3†	28 ± 3†	29 ± 3†
	+Betaine (2 mM)	27 ± 3†	29 ± 3†	30 ± 3†
+ Hypomethylator a	+DMSO (150 M)	80 ± 4†	84 ± 4†	89 ± 4†
	+ GSH (5 mM)	28 ± 3†	31 ± 3†	40 ± 4†
+ GSH synthesis stimulator a	+Trifluoperazine (15 M)	30 ± 3†	37 ± 4†	46 ± 5†
+GSH depleting agent a	+1-Bromoheptane	75 ± 4†	77 ± 4†	86 ± 4†

Hepatocytes (10^6^cells/mL) were incubated in Krebs–Henseleit buffer pH = 7.4 at 37 ^oC^. Mitochondrial membrane potential was determined as the difference in mitochondrial uptake of the rhodamine 123 between control and treated cells and expressed as fluorescence intensity unit [Andersson and Jones 1987]. GSH depleted hepatocytes were prepared as described by Khan and O’Brien [Khan and O'Brien 1991]. Values are expressed as mean ± SD of three separate experiments (n = 3).

PLGA NPs were not cytotoxic at all concentrations tested (data not shown).

a All these agents did not show any toxic effect on hepatocyte at concentrations used (data not shown).

* Significant difference in comparison with PLGA-DTX treated hepatocytes (P<0.05).

† Significant difference in comparison with DTX treated hepatocytes (P<0.05).

**Table 3:** Lysosomal membrane integrity changes during PLGA-DTX and DTX induced hepatocyte injury

**Treatment Group**	**Addition**	**% Acridine Orange Redistribution**		
		**Incubation Time**		
		**15 min**	**30 min**	**60 min**
Nano formulation of DTX	PLGA-DTX (3 nM)	52 ± 3	73 ± 4	89 ± 4
+Antioxidants^a^	+Mannitol (50 mM)	27 ± 3*	37 ± 4*	52 ± 3*
	+BHT (50 M)	32 ± 3*	36 ± 4*	38 ± 4*
+MPT pore-sealing Agents^a^	+Carnitine (2 mM)	35 ± 4*	37 ± 4*	39 ± 4*
	+Cyclosporin (2 M)	33 ± 3*	35 ± 4*	41 ± 4*
+Lysosomotropic Agents^a^	+Chloroquine (100 M)	18 ± 2*	19 ± 2*	20 ± 2*
	+Methylamine (30 mM)	19 ± 2*	22 ± 2*	23 ± 2*
	+ Monensin (10 M)	20 ± 2*	24 ± 2*	26 ± 3*
+ NADPH-P450 reductase inhibitor^a^	+Diphenyliodonium chloride (50 M)	13 ± 1*	14 ± 1*	15 ± 2*
+ CYP2E1 inhibitors^a^	+Phenylimidazole (300 M)	25 ± 2*	26 ± 3*	27 ± 3*
	+ 4-Methylpyrazole (500 M)	28 ± 3*	34 ± 3*	58 ± 3*
+ CYP3A4 inhibitors^a^	+ Troleandomycin (10 M)	73 ± 4*	77 ± 4*	83 ± 4*
	+ Ketoconazole (10 M)	72 ± 4*	76 ± 4*	84 ± 4*
+ Hepatoprotectant	+Silymarin (100 M)	18 ± 2*	19 ± 2*	20 ± 2*
+ Methyl Donors a	+Methionine (1 mM)	25 ± 3*	36 ± 4*	48 ± 5*
	+Folic acid (100 M)	20 ± 2*	34 ± 3*	40 ± 4*
	+Betaine (2 mM)	23 ± 2*	30 ± 3*	39 ± 4*
+ Hypomethylator a	+DMSO (150 M)	70 ± 3*	82 ± 4*	93 ± 5*
	+GSH (5 mM)	81 ± 4*	85 ± 4*	90 ± 5*
+ GSH synthesis stimulator a	+Trifluoperazine (15 M)	77 ± 4*	84 ± 4*	89 ± 4*
+GSH depleting agent a	+1-Bromoheptane	30 ± 3*	33 ± 3*	35 ± 3*
Free Drug	DTX (6 nM)	44 ± 4	67 ± 3	72 ± 4
+Antioxidants^a^	+Mannitol (50 mM)	29 ± 3 †	31 ± 3 †	35 ± 3†
	+BHT (50 M)	31 ± 3 †	33 ± 3 †	34 ± 3†
+MPT pore-sealing Agents^a^	+Carnitine (2 mM)	50 ± 3 †	56 ± 3 †	70 ± 3†
	+Cyclosporin (2 M)	43 ± 3 †	57 ± 4 †	61 ± 4†
+Lysosomotropic Agents^a^	+Chloroquine (100 M)	20 ± 2 †	22 ± 2 †	23 ± 2†
	+Methylamine (30 mM)	21 ± 2 †	24 ± 2 †	26 ± 3†
	+ Monensin (10 M)	22 ± 2 †	27 ± 3 †	33 ± 3†
+ NADPH-P450 reductase inhibitor^a^	+Diphenyliodonium chloride (50 M)	14 ± 1 †	16 ± 2 †	18 ± 2 †
+ CYP2E1 inhibitors^a^	+Phenylimidazole (300 M)	20 ± 2 †	24 ± 2 †	36 ± 3 †
	+ 4-Methylpyrazole (500 M)	24 ± 3 †	26 ± 3 †	37 ± 4 †
+ CYP3A4 inhibitors^a^	+ Troleandomycin (10 M)	60 ± 3 †	67 ± 3 †	72 ± 4 †
	+ Ketoconazole (10 M)	64 ± 3 †	68 ± 3 †	73 ± 3 †
+ Hepatoprotectant	+Silymarin (100 M)	37 ± 3†	58 ± 4 †	63 ± 3†
+ Methyl Donors a	+Methionine (1 mM)	22 ± 2 †	30 ± 3 †	45 ± 5 †
	+Folic acid (100 M)	20 ± 2 †	28 ± 3 †	40 ± 4 †
	+Betaine (2 mM)	26 ± 3 †	35 ± 4 †	47 ± 5 †
+ Hypomethylator a	+DMSO (150 M)	60 ± 3 †	71 ± 4 †	81 ± 4†
	+GSH (5 mM)	38 ± 4 †	41 ± 4 †	46 ± 5 †
+ GSH synthesis stimulator a	+Trifluoperazine (15 M)	31 ± 3 †	48 ± 5 †	57 ± 3†
+GSH depleting agent a	+1-Bromoheptane	77 ± 4 †	86 ± 4 †	93 ± 5 †

Hepatocytes (10^6^ cells/mL) were incubated in Krebs–Henseleit buffer pH = 7.4 at 37 ^oC^. Lysosomal membrane damage was determined as intensity unit of diffuse cytosolic green fluorescence induced by acridine orange following the release from lysosomes [Pourahmad et al. 2001]. GSH depleted hepatocytes were prepared as described by Khan and O’Brien [Khan and O'Brien 1991]. Values are expressed as mean ± SD of three separate experiments (n = 3).

PLGA NPs were not cytotoxic at all concentrations tested (data not shown).

a All these agents did not show any toxic effect on hepatocyte at concentrations used (data not shown).

* Significant difference in comparison with PLGA-DTX treated hepatocytes (P<0.05).

† Significant difference in comparison with DTX treated hepatocytes (P<0.05).

**Table 4:** Effect of antioxidants and ROS scavengers, lysosomotropic agents, MPT pore-sealing agents, methyldonor, and NADPH P450 reductase inhibitors on PLGA-DTX and DTX induced intracellular hepatocyte GSH decrease and extracellular GSSG increase

**Treatment Group**	**Addition**	**Int. GSH (M)** **3** **h**	**Ext. GSSG (M)** **3** **h**
Control	-	75 ± 4	11 ± 1
Nano formulation of DTX	PLGA-DTX(3 nM)	34 ± 3*	35 ± 4*
+Antioxidants^a^	+Mannitol (50 mM)	54 ± 3†	21 ± 3†
	+BHT (50 M)	50 ± 3†	22 ± 3†
+MPT pore-sealing Agents^a^	+Carnitine (2 mM)	51 ± 3†	23 ± 2†
	+Cyclosporin (2 M)	52 ± 3†	22 ± 3†
+Lysosomotropic Agents^a^	+Chloroquine (100 M)	54 ± 3†	24 ± 2†
	+Methylamine (30 mM)	53 ± 3†	23 ± 2†
	+ Monensin (10 M)	55 ± 3†	22 ± 3†
+ NADPH-P450 reductase inhibitor^a^	+Diphenyliodonium chloride (50 M)	58 ± 3†	21 ± 1†
+ CYP2E1 inhibitors^a^	+Phenylimidazole (300 M)	59 ± 3†	25 ± 3†
	+ 4-Methylpyrazole (500 M)	51 ± 3†	23 ± 3†
+ CYP3A4 inhibitors^a^	+ Troleandomycin (10 M)	21 ± 2†	92 ± 5†
	+ Ketoconazole (10 M)	20 ± 2†	95 ± 5†
+ Hepatprotectant	+Silymarin (100 M)	58 ± 3†	20 ± 2†
+ Methyl Donors a	+Methionine (1 mM)	57 ± 3†	29 ± 3†
	+Folic acid (100 M)	60 ± 3†	21 ± 3†
	+Betaine (2 mM)	59 ± 3†	24 ± 3†
+ Hypomethylator a	+DMSO (150 M)	23 ± 2†	41 ± 5†
Free Drug	DTX (6 nM)	43 ± 4*	30 ± 3*
+Antioxidants^a^	+Mannitol (50 mM)	52 ± 3†	23 ± 3†
	+BHT (50 M)	59 ± 3‡	24 ± 3‡
+MPT pore-sealing Agents^a^	+Carnitine (2 mM)	58 ± 3‡	29 ± 3‡
	+Cyclosporin (2 M)	56 ± 3‡	24 ± 2‡
+Lysosomotropic Agents^a^	+Chloroquine (100 M)	55 ± 3‡	23 ± 2‡
	+Methylamine (30 mM)	53 ± 3‡	23 ± 2‡
	+ Monensin (10 M)	54 ± 4‡	22 ± 3‡
+ NADPH-P450 reductase inhibitor^a^	+Diphenyliodonium chloride (50 M)	53 ± 4‡	20 ± 3‡
+ CYP2E1 inhibitors^a^	+Phenylimidazole (300 M)	58 ± 3‡	20 ± 3‡
	+ 4-Methylpyrazole (500 M)	56 ± 3‡	22 ± 3‡
+ CYP3A4 inhibitors^a^	+ Troleandomycin (10 M)	31 ± 3‡	82 ± 4‡
	+ Ketoconazole (10 M)	33 ± 3‡	86 ± 4‡
+ Hepatprotectant	+Silymarin (100 M)	48 ± 5‡	28 ± 4‡
+ Methyl Donors a	+Methionine (1 mM)	59 ± 3‡	22 ± 3‡
	+Folic acid (100 M)	60 ± 3‡	24 ± 3‡
	+Betaine (2 mM)	63 ± 3‡	23 ± 3‡
+ Hypomethylator a	+DMSO (150 M)	28 ± 3‡	70 ± 4‡

Hepatocytes (10^6^ cells/mL) were incubated in Krebs–Henseleit buffer pH = 7.4 at 37^ oC^ for 3.0 h following the addition of PLGA-DTX & DTX. Intracellular GSH and extracellular GSSG were flurimetrically determined as described by Hissin and Hilf [Hissin & Hilf 1976] are expressed as mean ± SD of three separate experiments (n= 3) P<0.05.

PLGA NPs were not cytotoxic at all concentrations tested (data not shown).

a All these agents did not show any toxic effect on hepatocyte at concentrations used (data not shown).

* Significant difference in comparison with control hepatocytes (P<0.05).

† Significant difference in comparison with PLGA-DTX treated hepatocytes (P<0.05).

‡ Significant difference in comparison with DTX treated hepatocytes (P<0.05).

**Figure1:**
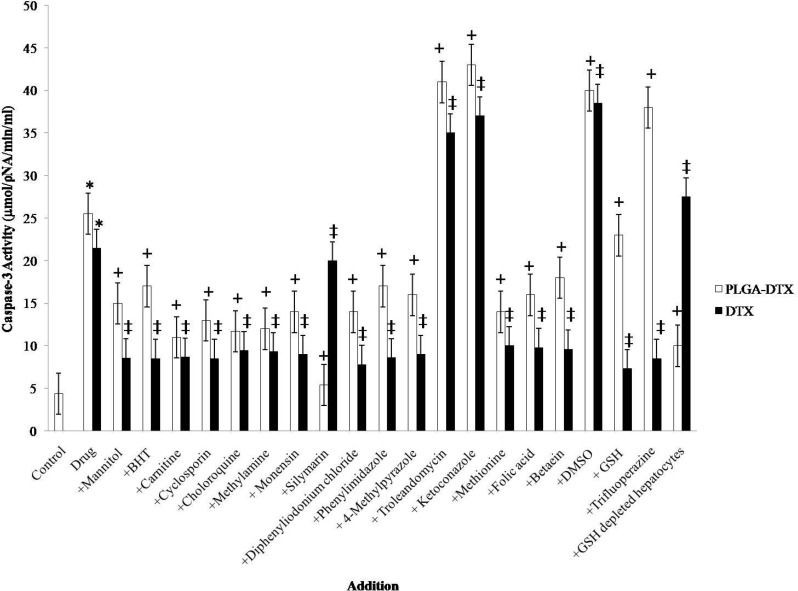
Effect of antioxidants and ROS scavengers, lysosomotropic agents, MPT pore-sealing agents, methyldonor, and NADPH P450 reductase inhibitors on PLGA-DTX and DTX induced hepatocyte Caspase-3 activation.

Hepatocytes (10^6^ cells/mL) were incubated in Krebs–Henseleit buffer pH 7.4 at 37 °C for 2 h following the addition of PLGA-DTX & DTX. Caspase-3 activity was determined by Sigma-Aldrich kit(26). The kit determines produced ρNA that is released from the interaction of caspase-3 and AC-DEVD-ρNA (peptide substrate). Values are expressed as mean ± SD of three separate experiments (n = 3).

PLGA NPs were not cytotoxic at all concentrations tested (data not shown).

^*^ Significant difference in comparison with control hepatocytes (P<0.05).

^+^ Significant difference in comparison with PLGA-DTX treated hepatocytes (P<0.05).

^‡^ **Significant difference in comparison with DTX treated hepatocytes (P<0.05).**

**Figure 2:**
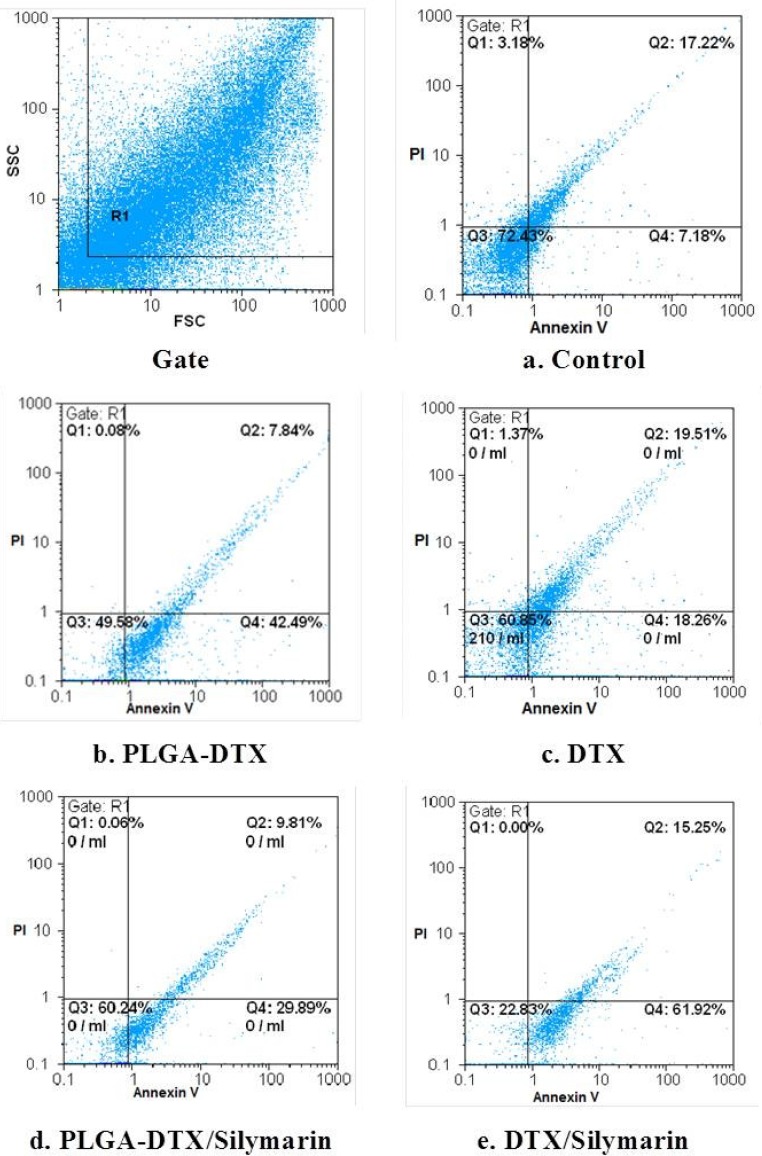
FCM of phospholipid redistribution: Annexin V/Propidium iodide assay. The technique was performed according to Vermes *et al*. (1995). The samples were analysed for green ﬂuorescence (FITC) and for red ﬂuorescence (PI) by ﬂow cytometry. The assay gives information about the numbers of vital (AV-/PI-) vs. apoptotic (AV+/PI-) cells, and provides also the number of secondary necrotic cells (AV+/PI+).a. Control. b. PLGA_DTX. c. PLGA-DTX/Silymarin. d. DTX. e. DTX/Silymarin. Hepatocytes (10^6^ cells/mL) were incubated in Krebs–Henseleit buffer pH 7.4 at 37 °C for 2 h following the addition of PLGA-DTX & DTX.

**PLGA NPs were not cytotoxic at all concentrations tested (data not shown).**

**Figure 3:**
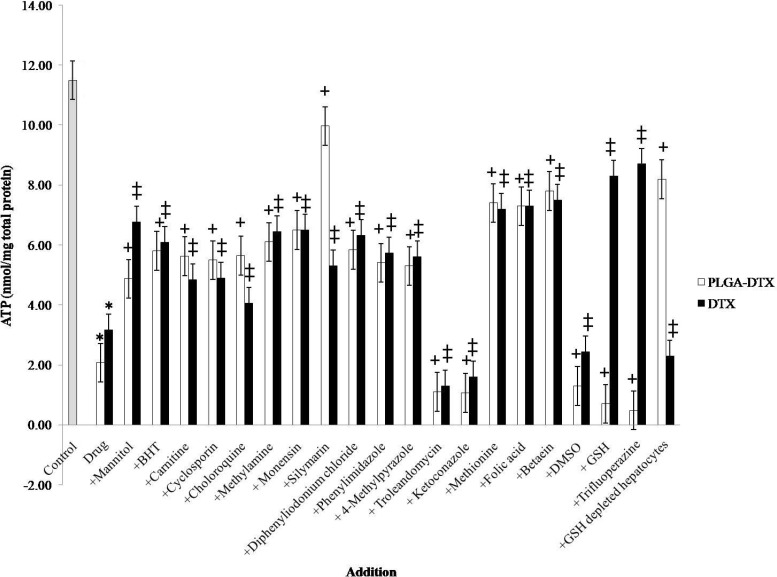
Effect of antioxidants and ROS scavengers, lysosomotropic agents, MPT pore-sealing agents, methyldonor, and NADPH P450 reductase inhibitors on PLGA-DTX and DTX induced hepatocyte ATP depletion.

Hepatocytes (10^6^ cells/mL) were incubated in Krebs–Henseleit buffer pH 7.4 at 37 °C for 2 h following the addition of PLGA-DTX & DTX.Caspase-3 activity was determined by Sigma-Aldrich kit (Sakahira H 1998). The kit determines produced ρNA that is released from the interaction of caspase- 3 and AC-DEVD-ρNA (peptide substrate). Values are expressed as mean ± SD of three separate experiments (n = 3).

PLGA NPs were not cytotoxic at all concentrations tested (data not shown).

^*^ Significant difference in comparison with control hepatocytes (P<0.05).

^+^Significant difference in comparison with PLGA-DTX treated hepatocytes (P<0.05).

^‡^ **Significant difference in comparison with DTX treated hepatocytes (P<0.05).**

As shown in Table 2. both DTX and PLGA-DTX induced a rapid decline in mitochondrial membrane potential, which was prevented by antioxidants and hydroxyl radical scavengers (BHT & mannitol) indicating that the decline of mitochondrial membrane potential was caused by ROS formation. In addition, lysosomotropic agents (methylamine, chloroquine, monensin) inhibited decline of mitochondrial membrane potential similar to PLGA-DTX and methyl donors (methionine, betaine & folic acid) inhibited decline of mitochondrial membrane potential in both agents.

On the other hand, as shown in Table 2. depleting hepatocyte methyl groups by hypomethylator such as DMSO significantly increased both DTX and PLGA-DTX collapse of mitochondrial membrane potential. All the pretreatment did not show any significant changes on the mitochondrial membrane potential at concentrations used when incubated alone in hepatocytes (data not shown).

When hepatocyte lysosomes were loaded with acridine orange (a lysosomotropic fluorescent probe), a significant release of acridine orange into the cytosolic fraction ensued within 60 min of incubation with both DTX and PLGA-DTX, indicating a severe damage to lysosomal membrane (Table 3). Both DTX and PLGA-DTX-induced acridine orange release was prevented by antioxidants and radical scavengers following addition of both compounds (BHT & mannitol), and MPT pore-sealing agents (cyclosporin & carnitine). DTX-induced acridine orange release was inhibited by GSH and GSH synthesis stimulator, trifluoperazine. On the other hand, PLGA-DTX-induced acridine orange release was potentiated by GSH and trifluoperazine (Table 3). All these substances did not show any significant effect on hepatocytes acridine orange release at concentrations used (data not shown). Depleting hepatocyte GSH beforehand potentiated DTX-induced acridine orange release; however, it prevented PLGA-DTX-induced acridine orange release (Table 3).

Both DTX and PLGA-DTX increased apoptosis signaling final mediator caspase-3 activity (Figure 1). Increased caspase-3 activity was prevented by MPT pore-sealing agents (carnitine, cyclosporin), and lysosomotropic agents (chloroquine, monensin, methylamine).

Most of the DTX and PLGA-DTX-induced GSH depletion could be attributed to ROS mediated oxidation of GSH to GSSG and subsequent expulsion of GSSG from hepatocytes (Table 4). Hydroxyl radical scavengers and antioxidants (Mannitol & BHT), MPT pore-sealing agents (carnitine & cyclosporin), lysosomotropic agents (chloroquine, monensin, methylamine) significantly (P < 0.05) prevented DTX and PLGA-DTX-induced GSH depletion (Table 4). All these reagents did not show any significant effect on both intracellular GSH decrease and extracellular GSSG increase at concentrations used (data not shown). To check out the involvement of cytochrome P450s on DTX and PLGA-DTX cytotoxicity mechanisms, we evaluated the effect of NADPH P450 reductase inhibitor, (diphenyliodonium chloride) on all the measured cytotoxicity markers. This agent significantly (P < 0.05) prevented both DTX and PLGA-DTX-induced cytotoxicity, ROS generation, decline of mitochondrial membrane potential, acridine orange release, increase of caspase-3 activity, and GSH depletion. diphenyliodonium chloride did not demonstrate any significant effect on the mentioned cytotoxicity markers at concentration used (data not shown).

The FSC-SSC histograms obtained from the flow cytometric analyses of fresh hepatocytes for 120 min are shown in Figure 2. Staining control group with a combination of FITC-conjugated annexin V and PI showed that great proportion of cells remains intact (Q3 = 72.43% in Figure 2a). 

As shown in Figure 2b the percentage of early apoptotic cells (Q4), and late apoptotic or necrotic cells (Q2) are quite different in hepatocytes which incubated with EC_50_2h (2 h of PLGA-DTX and DTX. As illustrated in figures 2d and 2e these proportion entirely changed when hepatocytes pretreated with silymarin. 

Figure 3. illustrated that, both DTX and PLGA-DTX can cause severe decline in intracellular ATP and the inhibitors such as lysosomotropic agents (chloroquine, methylamine, monensin), GSH synthesis stimulator (trifluoperazine) and silymarin do not have the same effect on free docetaxel and its nano formulation.

## Discussion

We have shown that PLGA-DTX was much more cytotoxic toward isolated rat hepatocytes than DTX alone. The EC_50_2h (2 h) concentration found for PLGA-DTX (i.e., 50% membrane lysis in 2 h) was 3 nM, while it was 6 nM for free docetaxel. As shown in Table 1. both DTX and PLGA-DTX significantly (P < 0.05) increased hepatocyte membrane lysis as determined by trypan blue exclusion test. 

Generation of ROS has been widely viewed as a common pathway of cell death from a variety of chemical agents (29). Present work showed that, when hepatocytes were incubated with both DTX and PLGA-DTX at this EC_50_2h (in 2 h) concentration, reactive oxygen species formation determined by the oxidation of dichlorofluorescin (DCFH) to dichlorofluorescein (DCF) was also significantly (P < 0.05) increased. DTX and PLGA-DTX-induced cytotoxicity and reactive oxygen species formation were prevented by radical scavengers and antioxidants (mannitol and BHT), MPT pore sealing agents (carnitine and cyclosporine), lysosomtropic agents (methylamine, chloroquine, monensin) as well as by NADPH P450 reductase inhibitor (diphenyliodoniumchloride) and CYP2E1 inhibitors (phenylimidazole, methylpyrazole) (Table 1).

The results presented here showed that in intact hepatocytes, both DTX and PLGA-DTX induced a decrease of mitochondrial membrane potential which was prevented by antioxidant and radical scavengers, indicating that mitochondrial membrane potential decrease was a consequence of reactive oxygen species formation (Table 2). 

In addition, DTX and PLGA-DTX induced lysosomal membrane leakiness, since acridine orange was significantly (P<0.05) released from lysosome to cytosol following incubation of DTX and PLGA-DTX in the acridine orange-loaded hepatocytes. Hepatocyte lysosomal leakiness occurred within 60 min following addition of DTX and PLGA-DTX, before toxicity ensued (Table 3). As a consequence, the deadly digestive lysosomal enzyme (proteases/phospholipases) released to the cytosol. The release of this could then be the ultimate cause of the induction of cell death process. 

We have shown that lysosomal leakiness prevents by hepatocyte lysosomotropic agents (methylamine, chloroquine and monensin) (Table 3). These results suggest that DTX and PLGA-DTX induced hepatocyte toxicity involves oxidative stress and formation of reactive oxygen species.

Caspase-3 is the most notable among the executioner caspases. The major pathway for caspase-3 activation is MPT pore opening and release of cytochrome c from mitochondria. GSH depletion and lysosomal membrane leakage could also accelerate and exacerbate the MPT pore opening and cytochrome c release. In view of that, we measured the caspase-3 activity which was augmented in hepatocytes when incubated with DTX or PLGA-DTX. 

Our results showed that PLGA-DTX and free docetaxel caused hepatocyte GSH depletion. However, these drugs showed different effects when incubated with GSH or GSH-depleted hepatocytes. GSH and GSH synthesis stimulator, trifluoperazine, prevented DTX-induced cytotoxicity, ROS generation, mitochondrial membrane damage, lysosomal membrane damage, ATP depletion and caspase-3 activity. In addition, depleting hepatocyte GSH beforehand potentiated hepatocyte susceptibility to DTX-induced cytotoxicity, ROS generation, mitochondrial membrane damage, lysosomal membrane damage, ATP depletion and caspase-3 activity. 

On the other hand, cytotoxicity, ROS generation, mitochondrial membrane damage, lysosomal membrane damage, ATP depletion and caspase-3 activation induced by PLGA-DTX was interestingly potentiated by GSH and trifluoperazine. Besides, depleting hepatocyte GSH beforehand prevented the hepatocytes against PLGA-DTX-induced cytotoxicity, ROS generation, mitochondrial membrane damage, lysosomal membrane damage, ATP depletion and caspase-3 activity.

In this study, we evaluated the effects of CYP450s on DTX and PLGA-DTX toxicity. Our results showed that NADPH P450 reductase inhibitor, diphenyliodonium chloride, prevented both DTX and PLGA-DTX-induced cytotoxicity, which revealed that these compounds were activated by CYP450 mediated metabolisim. However, it was already proved that CYP450s have a major role in DTX metabolism. Our results showed that CYP3A4 inhibitors (ketoconazole, troleandamycin) exacerbated the toxic effect of both DTX and PLGA-DTX, but CYP2E1 inhibitors (phenylimidazole, methylpyrazole) prevented drug-induced toxicity. These findings confirmed that CYP2E1 is directly involved in metabolic activation of both PLGA-DTX and DTX while CYP3A4 acts as a detoxification system.

We also investigated the protective effect of silymarin (30-33) against DTX and PLGA-DTX in isolated rat hepatocytes. The interesting findings were that silymarin could significantly diminish cytotoxicity of PLGA loaded docetaxel but not free DTX (Table 1). Silymarin also prevented apoptosis (Figure 2) ROS generation, mitochondrial membrane damage, lysosomal membrane damage, ATP and GSH depletion induced by PLGA-DTX but not free docetaxel (Table 1-3). These effects must be the result of multi characteristic functions of this herb such, inhibition of hepatic cytochrome P450s (CYPs), acting as free radical scavenger and also inhibitor of drug resistance protein in cell membrane such as P-gp efflux pump present in the hepatocyte (34), leading to considerable enhancement in the bioavailability of docetaxel in hepatocytes. These results suggest that nano formulation of docetaxel (unlike free drug), perhaps doesn’t have significant affinity to P-gp efflux pump and achieve much more reliable concentration than free docetaxel in hepatocytes and thus inhibitory effect of silymarin doesn’t affect PLGA-DTX cellular bioavailability. 

We have shown that lysosomal leakiness is prevented by the hepatocyte lysosomotropic agents (lysosomal inactivators) methylamine, chloroquine and monensin (Table 3). These results suggest that DTX and PLGA-DTX-induced hepatocyte toxicity involves oxidative stress and formation of H_2_O_2_ which is a mobile reactive oxygen species which could easily diffuse inside the lysosomes and start the intralysosomal Fenton reaction with Fe^2+^/Fe^3+^ leading to hydroxyl radical formation and subsequent lysosomal membrane damage. 

Quite unexpectedly, mitochondrial pore sealing agents (carnitin & cyclosporin) significantly (P < 0.05) inhibited release of acridine orange in hepatocytes treated with PLGA-DTX and also lysosomotropic agents (methylamine, chloroquine and monensin) prevented mitochondrial membrane damage merely in nano formulation. All these interesting events brought us to a conclusion that there is likely a cross-talk between mitochondria and lysosomes in PLGA-DTX toxicity but not in DTX.

We have also shown that both DTX and PLGA-DTX induced cytotoxic alterations were potentiated by preincubating with hypomethylating agent (DMSO). On the other hand, natural methyl donors (methionine, betaine and folic acid) prevented both DTX and PLGA-DTX-induced hepatocyte lysis, ROS formation, mitochondrial membrane potential decrease, lysosomal membrane leakiness GSH and ATP depletion. As a result, we suggested that both DTX and PLGA-DTX detoxification pathway in mammalian cells is surely metabolic methylation.

It is well known that glutathione (GSH) is a major antioxidant as well as redox and cell signaling regulator. We demonstrated that depleting hepatocyte GSH beforehand increased hepatocyte sensitivity to DTX-induced cytotoxicity, ROS formation, mitochondrial membrane damage, lysosomal membrane damage and caspase-3 activity. These effects could be attributed to the antioxidant effects of GSH. On the other hand, cytotoxicity, ROS generation and other toxic end points such as mitochondrial membrane potential decrease, lysosomal membrane damage, and caspase-3 activation induced by PLGA-DTX were all potentiated by GSH and trifluoperazine, a GSH synthesis stimulator. This could be attributed to the role of GSH in PLGA-DTX bioactivation (Table 4).

Several experiments suggest that cellular ATP level is an important determinant for cell death, either by apoptosis or necrosis. It is proven that cells need a particular ATP level to stay alive, so if the amount of ATP falls below this level apoptosis or necrosis could occur (35, 36). Our results also revealed that both DTX and PLGA-loaded DTX could reduce intracellular ATP level; and the effect of PLGA-DTX but not DTX was significantly inhibited by silymarin and GSH depleting agent (1-bromoheptane). All these data were consistent with our other findings about the role of depletion of GSH pool and oxidative stress in PLGA-DTX-induced toxicity.

In various investigations, researchers showed that cellular mortality of PLGA nanoparticles containing docetaxel was higher than free drug in various cancerous cell lines (15, 37). Our results are in accordance with this work. However, we have also shown this formulation could also cause severe toxic effects in normal cells such as hepatocytes. Thus it is suggested that future administration in cancer patients should be carried out with caution.
